# Proanthocyanidin Attenuation of Oxidative Stress and NF-****κ****B Protects Apolipoprotein E-Deficient Mice against Diabetic Nephropathy

**DOI:** 10.1155/2013/769409

**Published:** 2013-08-18

**Authors:** Abdulrahman L. Al-Malki, Ahmed Amir Radwan Sayed, Haddad A. El Rabey

**Affiliations:** ^1^Department of Biochemistry, Faculty of Science, King Abdulaziz University, P.O. Box 80203, Jeddah 21589, Saudi Arabia; ^2^Chemistry Department, Faculty of Science, Minia University, EL-Minia 61519, Egypt; ^3^Genetic Engineering and Biotechnology Institute, Minufiya University, Sadat City, Egypt

## Abstract

Hyperlipidemia and hyperglycemia result in oxidative stress and play a major role in the development of diabetic nephropathy (DN). We explored the effects of proanthocyanidin (PA) on the induction and progression of DN in apolipoprotein E-deficient mice. Diabetes Mellitus was induced in ten-week-old male apoE^−/−^mice using streptozotocin (STZ). Mice were fed with a high-fat diet in presence or absence of PA. PA treatment significantly reduced the high cholesterol levels, restored renal functions, and reduced albuminuria in the PA-treated diabetic mice compared with the diabetic untreated mice. In addition, the glomerular mesangial expansion in the diabetic mice was attenuated as a result of PA supplementation. Moreover, PA treatment restored the elevated levels of MDA and CML and the reduced activity of SOD and GSH in the diabetic mice. Furthermore, PA feeding reduced the activation and translocation of NF-**κ**B to the nucleus compared with the diabetic untreated animals. Reduction of NF-**κ**B activation resulted in the attenuation of the expression of IL-6, TGF**β**, and RAGE which protected PA-treated mice against DN. The renoprotective effects of PA were found to be time independent regardless of whether the dietary feeding with PA was started pre-, co-, or post-STZ injection. In conclusion, part of the beneficial effects of PA includes the disruption of the detrimental AGE-RAGE-NF**κ**B pathways.

## 1. Introduction

Diabetes mellitus (DM) is considered as the leading cause of end-stage renal disease. The relative risk for cardiovascular diseases is 10 folds higher for type-1 diabetic patients with nephropathy compared with those without diabetic nephropathy (DN) [1]. Proteinuria, which was considered an indicator of underlying DN, usually worsens with progression of diabetic kidney disease. This progression is characterized by declining glomerular filtration rate and kidney structural changes, including thickening of the basement membranes, mesangial sclerosis, and arteriolar hyalinosis.

Hyperglycemia is a major cause for the increased glycation of proteins and lipids that, in turn, enhances the generation of reactive oxygen species (ROS) [2–4]. Thus, diabetes mellitus is usually accompanied by an increased ROS production and impaired antioxidant defense mechanisms leading to increased oxidative stress. ROS interact with the free amino and sulfhydryl groups of proteins forming Amadori products which further modified to form advanced glycation endproducts (AGEs) specially carboxymethyl lysine (CML). The formed AGEs bind to their receptors (AGE-receptors) on the cell membrane resulting in the activation of the nuclear factor kaba B (NF-*κ*B). NF-*κ*B is found in the cytoplasm of the normal nonactivated cells binding to its inhibitor (I-*κ*B). As a result of increased AGE production and interaction of AGE with RAGE, NF-*κ*B dissociates from I-*κ*B and migrates to the cell nucleus. Once in the nucleus, it stimulates the transcription of its controlled genes. The genes are inflammatory genes like interleukin-6, IL-1*β*, RAGE, TNF*α*, and others. Overexpression of these inflammatory genes plays a key role in the pathogenesis of diabetic complications especially diabetic nephropathy [5–9].

In diabetic patients with suboptimal glycemic control, hyperlipidemia may develop. In addition, even in subjects with optimal glycemic control, the abnormal lipid profile that is potentially atherogenic may exist. In diabetes mellitus, hyperlipidemia is considered an independent and major determinant of progression of renal disease [10]. In this regard, an association between high consumption of saturated fat and albuminuria in type-1 diabetic patients has been reported [11].

Proanthocyanidin (PA) are polyphenolic compounds, which are derivatives of flavan-3-ol flavonoids. They are mainly composed of dimers, oligomers, and polymers of (+)-catechin, (−) epicatchin, and their gallic acid esters. Their degree of polymerization is generally distributed between 2 and 15. The wide presence of PAs in plants makes them an important part of the human diet [12].

PAs have been reported to show various beneficial properties. In addition to their free radical scavenging and antioxidant activity [13], PAs have been reported to have antibacterial, antiviral, anticancer, anti-inflammatory, anti-allergic, and vasodilator actions [14]. Moreover, PAs were found to attenuate diabetic nephropathy in rats [15] and cisplatin-induced nephrotoxicity [16]. Furthermore, PA was found to inhibit lipid peroxidation, platelet aggregation, capillary permeability, and fragility and to affect enzyme systems including phospholipase A_2_, cyclooxygenase, and lipoxygenase [14]. The free radical scavenging activity of PAs has been well documented and commanded the most attention [14]. *In vivo *studies have shown that grape seed PA extract is a better free radical scavenger and inhibitor of oxidative tissue damage than vitamin C, vitamin E succinate, vitamin C and vitamin E succinate combined, and beta carotene [14]. 

The present study examined the effect of dietary PA supplementation on diabetes-mellitus-induced kidney damage in apoE−/mice, an animal model previously demonstrated to have a series of pathological conditions including dyslipidemia and atherosclerosis. Hyperlipidemia per se is associated with the development of early renal lesions in apoE−/mice [17]. Mice were rendered diabetic by intraperitoneal administration of low doses of STZ over 5 days, an established approach for inducing type-1 diabetes mellitus [18]. Given the previous features, STZ-induced diabetic apoE−/mice fed high-fat diet were used in our studies to accelerate DN and probe the role of dyslipidemia in DN development [19].

## 2. Materials and Methods

### 2.1. Experimental Design

All of the animal experiments were carried out under protocols approved by the Institutional Animal House of the University of King Abdulaziz at Jeddah, Saudi Arabia. Male apoE−/mice on C57BL/6J genetic background (14 weeks old) were divided into four groups, both control and diabetic groups, without or with PA supplementation. Diabetes mellitus was induced in the mice by intraperitoneal injections of STZ at a dose of 40 mg/kg bodyweight in citrate buffer, pH 4.5 (Sigma, St. Louis, MO, USA) for 5 days [18]. Animals of the control group received only citrate buffer. If the blood glucose concentration of a mouse is higher than 250 mg/dL 72 hours after the STZ injection, it is considered as a diabetic animal. Mice with blood glucose lower than 250 mg/dL were excluded from the experiment. Mice of all groups were housed in cages and received normal diet and tap water *ad libitum* in a constant environment (room temperature 25 ± 2°C, room humidity 50 ± 5%) with a 12 h light, 12 h dark cycle until being changed to a synthetic high-fat (HF) diet containing 21% (w/w, or 45% calories) fat, 0.05% cholesterol, 20% sucrose (Research Diets, Inc., New Brunswick, NJ, USA) [19] with or without 500 mg/kg PA purchased from Bioline Company, England [15]. Bodyweights and blood glucose levels were measured regularly. At the end of the experiment (12 weeks after induction of diabetes), animals were sacrificed using ether anaesthesia. Blood was collected and centrifuged, and serum was stored at −80°C and used for the assay of cytokines and biochemical and oxidative stress markers. Kidneys were removed and rinsed with cold PBS and then weighed.

Kidneys from each mouse were divided into several parts. The first part was used to prepare the kidney homogenate as discussed later. This homogenate was used for assaying the oxidant/antioxidant parameters as well as CML and IL-6 in the mice kidney. The second part was used for isolation of total RNA and DNA from the kidney cortex. The third part was used for preparation of the nuclear and cytoplasmic extracts for EMSA and Western blot analyses. The fourth part was used for histopathological examination.

### 2.2. Blood Sampling and Analysis

The diagnostic kits for determinations of levels of glucose, creatinine (Cr), blood urea nitrogen (BUN), sodium, potassium, cholesterol, and triglycerides were purchased from BioSystem (Barcelona, Spain). Serum cytokines and antioxidant markers as well as AGEs were assayed using commercially available kits. All analyses were performed in accordance with the manuals provided by the manufacturer.

### 2.3. Kidney Homogenate Preparation

Part of the kidney was cut into small pieces and washed by cold phosphate-buffered saline (PBS). Furthermore, it was ground in a homogenization buffer {0.05 M Tris-HCl pH 7.9, 25% glycerol, 0.1 mM EDTA, and 0.32 M (NH_4_)_2_SO_4_} containing a protease inhibitor tablet (Roche, Germany). The solution was sonicated in an ice bath for 15 seconds followed by centrifugation at 12000 rpm, 4°C for 5 minutes. The supernatant was aliquoted, stored at −80°C, and assayed for protein concentration using BCA kit (Pierce, Rockford, IL, USA) using albumin diluted in a lysis buffer as standard [20]. The homogenate was used for the determination of reduced glutathione (GSH), level of lipid peroxidation (MDA), concentration of *N*ε**-carboxymethyl lysine (CML), activity of superoxide dismutase (SOD), and level of IL-6.

### 2.4. Determining Enzymatic Activities

The activities of total SOD as well as the concentrations of MDA and GSH in the kidney homogenate were determined using commercially available kits from BioVision Research Products (Linda Vista Avenue, Pasadena, CA, USA) according to the methods described by Nishikimi et al. [21]; Mekheimer et al., Ohkawa et al. [22, 23]; and Moron et al. [24], respectively. 

### 2.5. Determination of IL-6

IL-6 concentration in the serum and in the kidney homogenate was determined by an ELISA kit. The ELISA for determination of IL-6 was performed using a commercially available kit from R&D (Mannheim, Germany) according to the instructions of the manufacturer. 

### 2.6. Analysis of Urine Parameters

Urine samples were collected by placing the mice in individual metabolic cages for 24 h before diabetes mellitus had been induced and the day before the end of experiment. The urine albumin concentration was determined with an ELISA kit (Nephrat II, Exocell, Philadelphia, PA, USA), and the concentration of Cr in pooled urine samples was determined by the commercial assay kit. All analyses were performed in accordance with the manuals provided by the manufacturers.

### 2.7. Measurement of Urinary and Renal 8-Hydroxy-2′-deoxyguanosine

Urinary 8-hydroxy-2′-deoxyguanosine (8-OHdG) levels were determined using an ELISA kit from Genox Corporation (Baltimore, MD, USA) according to the method of Matsubasa et al. [25] and were corrected by using individual urine creatinine concentrations. Extraction of the renal DNA was performed using a DNA extraction kit (Promega, Germany) following the manufacturer's protocol. The genomic DNA samples from kidney tissues were also used for the determination of 8-OHdG using the competitive ELISA kit.

### 2.8. Assay of Serum and Renal CML

The supernatant of the kidney homogenate was tested for CML using the anti-CML mouse autoantibody ELISA kit which employs the semiquantitative enzyme immunoassay technique. The ELISA kit for CML was provided by Roche Diagnostics (Mannheim, Germany). The absorbance of the resulting yellow product was measured at 450 nm [26–28].

### 2.9. Histopathological Examination

Renal tissues were collected after animal sacrifice, fixed in 10% formalin, processed routinely, and embedded in paraffin. 5 *μ*m thick sections were prepared and stained with periodic acid-Schiff (PAS). Glomerular histopathological changes and mesangial lesions were scored in terms of the glomerular mesangial expansion (increase in the mesangial matrix). Mesangial matrix expansion (MME) was derived from the estimation of 100 glomeruli of 5 nondiabetic and 5 diabetic mice per dietary group [29].

### 2.10. Reverse Transcription Polymerase Chain Reaction

Total RNA of mice in different groups was isolated from kidney cortex by using TRIzol reagent (Invitrogen, Darmstadt, Germany) according to the manufacturer's protocol. Gene expressions were determined using real-time quantitative reverse transcription polymerase chain reaction. **β**-actin was considered as a reference gene. The sequences of the used primers were as follows.

*Nephrin* sens 5′-ACT ACG CCC TCT TCA AAT GCA-3′, antisens 5′-TCG AGG GCC TCA TAC CTG AT-3′; *TGF*β*1* sens 5′-TGG AGC AAC ATG TGG AAC TC-3′, antisens 5′-GTC AGC AGC CGG TTA CCA-3′; *RAGE* sens 5′-CAC AGC CCG GAT TG-3′, antisens 5′-GCT GTA GCT GGT GGT CAG AAC A-3′; *SOD2* sens 5′-ACT GAA GTT CAA TGG TGG GG-3′, antisens 5′-GCT TGA TAG CCT CCA GCA AC-3′; **β*-actin* sens 5′-GTG CTA TGT TGC TCT AGA CTT CG-3′, antisens 5′-ATG CCA CAG GAT TCC ATA CC-3′.

### 2.11. Western Blot Analysis

Cytoplasmic and nuclear fractions of the mice kidneys were separated as described [7]. In brief, cytoplasmic and nuclear extracts were separated by adding 200 *μ*L buffer A (10 mM HEPES-KOH, pH 7.9 at 4°C, 1.5 mM MgCl_2_, 10 mM KCl, 0.5 mM DTT, and 0.2 mM PMSF) to the kidney, mixed well for 30 seconds, incubated on ice for 15 minutes, mixed again, and centrifuged at 13000 rpm. for 5 minutes. The supernatant contained the cytoplasmic extract and was transferred to a fresh tube and kept at –80°C. To the pellet, 50 *μ*L buffer C (20 mM HEPES-KOH, pH 7.9 at 4°C, 25% glycerol, 420 mM NaCl, 1.5 mM MgCl_2_, and 0.2 mM EDTA) was added, vortexed for 30 seconds, incubated in ice for 40 minutes and vortexed every 10 minutes, and centrifuged at 13000 rpm for 5 minutes. The supernatant contains the nuclear extract and was transferred to a fresh tube and kept at –80°C.

Western blot was performed as described. Proteins were separated on a polyacrylamide gel and transferred to a nitrocellulose membrane (Amersham Pharmacia, Braunschweig, Germany). Membranes were blocked with nonfat milk, incubated with primary antibodies directed against NF-*κ*B-p65 overnight at 4°C. After washing three times with PBS, the secondary antibody (horseradish peroxidase-coupled rabbit IgG) was added, and incubation was continued for 60 minutes. After washing 5 times, the immunoreactive proteins were detected with the ECL-Western blot system (Amersham Pharmacia, Braunschweig, Germany) and subsequent autoradiography for 2 minutes. All experiments were performed three times [7, 9].

### 2.12. Statistical Analysis

All of the biochemical assays were carried out in duplicates. Statistical analysis was performed with JMP software (SAS Institute, Cary, NC, USA). Diabetes mellitus effect, PA effect, and their interaction were analyzed using the two-way ANOVA, and Tukey-Kramer HSD test was applied for post hoc pairwise comparisons. Time effect was analyzed by MANOVA for repeated measurement.

## 3. Results

Results obtained showed thatthe diabetic untreated mice suffer from loss of bodyweight, but the diabetic mice treated with PA were able to keep their bodyweights (*P* < 0.01; Table 1). Moreover, PA treatment markedly slowed the gain of weight induced by HF diet in the nondiabetic mice, although the two groups received the daily dietary intake with or without PA (Table 1).

Although both bodyweight loss and hyperglycemia were reduced in mice within the pre-STZ group, the obtained data from mice of the three dietary groups were not markedly affected by the time of PA supplementation.

### 3.1. Markers of Oxidative Stress

Diabetes mellitus results in a significant increase of oxidative stress markers, as reflected by the reduction in serum and renal cortex GSH and increase of MDA and AGEs. Dietary PA administration ameliorated all of these changes (Tables 1 and 2). A similar effect on the urinary 8-isoprostane was obtained. The urinary 8-isoprostane was significantly increased as a result of diabetes, while dietary PA administration attenuated these elevated levels (Table 1). Total SOD activity in kidney homogenate was decreased as a result of diabetes mellitus. Treatment with dietary PA partially restored the reduced SOD activity (Table 2). 

### 3.2. Activation of NF-*κ*B in the Mice Kidney

**Figure 1:**
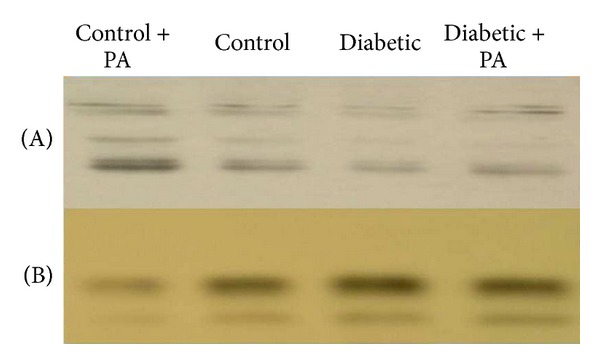
NF-*κ*B activation in the rat kidney: Western blot of rat kidneys of control rats, control rats + PA, diabetic rats, and diabetic rats treated with PA. Cytoplasmic (A) and nuclear (B) extracts were obtained as described in the Materials and Methods section.

As a result of oxidative stress and formation of AGE in the diabetic mice, we examined the activation of NF-*κ*B in the mice kidneys using both EMSA and Western blot analysis. Data obtained from EMSA showed a marked increase in the activity of NF-*κ*B-p65 compared with the control group. Treatment of diabetic mice with PA resulted in a significant reduction of NF-*κ*B-p65 activity as shown in Figure 1. Using Western blot analysis, the translocation of NF-*κ*B-p65 from the cytoplasm into the nucleus was observed. Feeding of the diabetic mice with PA normalizes these disorders.

### 3.3. Plasma Cytokines

Previous data [5, 7, 9] showed that activation of NF-*κ*B results in the overexpression of cytokines like IL-6, TNF*α*, TGF*β*1, and RAGE. In order to examine this hypothesis, we assayed the levels of IL-6 and TNF*α* in the mice serum. The levels of IL-6 in the diabetic mice were significantly increased compared with the control mice. Dietary feeding with PA significantly reduced the increase of IL-6 compared with the diabetic untreated mice. In contrast, there were no significant differences in the levels of TNF*α* in the diabetic and nondiabetic mice (Table 1).

### 3.4. Expression of Some Genes in the Kidney

**Figure 2:**
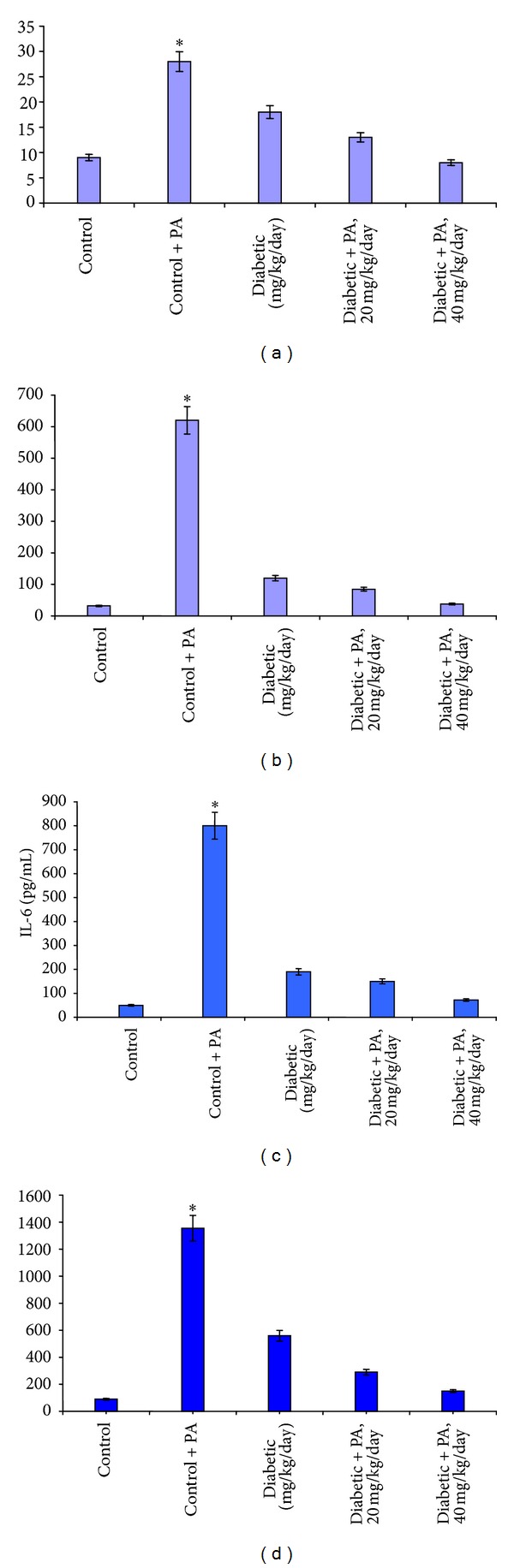
Nephrin mRNA expression (a), TGF*β*1 (b), RAGE (c) and SOD2 (d) expressions were measured using ELISA kit. Kidneys and sera were obtained from all groups treated with saline and PA and mice pretreated with different concentrations of proanthocyanidin. Mean values ±SD are shown. The asterisk (*) values are different (*P* < 0.05) as compared with control group.

Activation of NF-*κ*B results in the overexpression of the inflammatory genes as previously described [7, 9]. The expression of nephrin and SOD2 genes in the kidney cortex of the diabetic untreated mice was downregulated. PA supplementation resulted in the upregulation of the expression of the two genes (Figures 2(a) and 2(d)). In contrast, the expression of the AGE receptor (RAGE) and the transforming growth factor *β*1 (TGF*β*1) in the kidney cortex was upregulated due to STZ treatment and diabetes mellitus. Treatment of the diabetic mice with PA restored their expression near the control group as shown in Figures 2(b) and 2(c).

### 3.5. Renal Damage

All of the diabetic mice suffered from polyuria. PA treatment improved to some extent this disorder, although the effect was not statistically significant (Table 1). Kidney weight over body weight in the diabetic untreated mice was 40% higher than that of nondiabetic mice (*P* < 0.05); this resulted from the reduced body weight of the diabetic mice. As a result of dietary administration of PA, the reduction in the KW/BW ratio was reduced (Table 1). As a result of diabetes mellitus and the earlier forms of the diabetic kidney disease, the daily albuminuria excretion was clearly elevated in diabetic mice. PA supplementation significantly attenuated albuminuria (Table 1).

**Figure 3:**
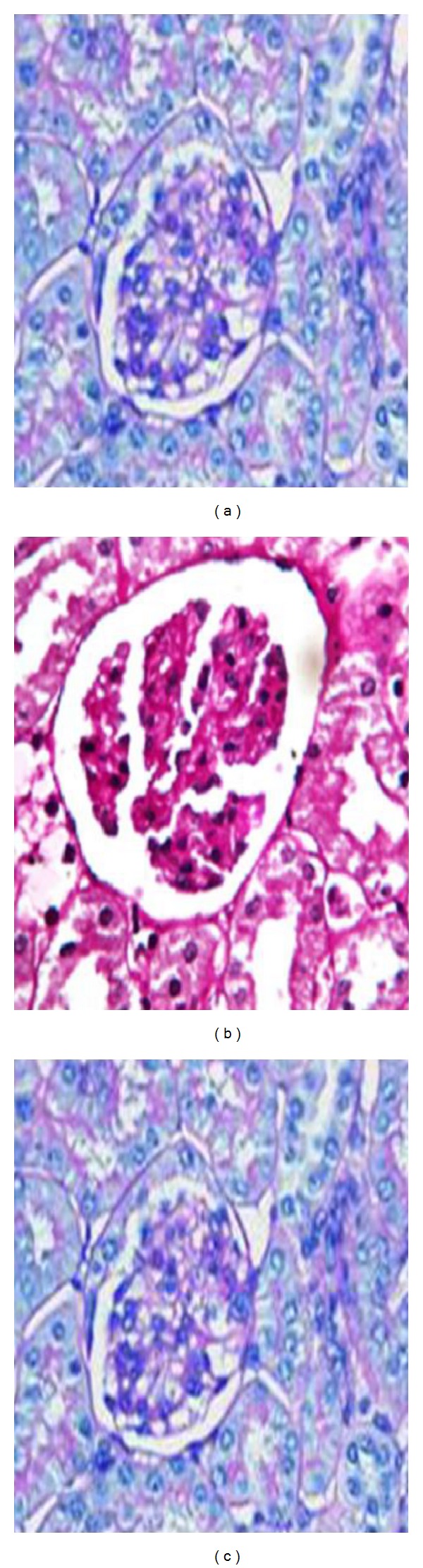
Mesangial expansion and ultrastructural changes in diabetic apoE−/mice after 12 weeks after induction of diabetes mellitus. (a) Nondiabetic mice without PA. (b) Diabetic mice without PA. (c) Diabetic mice with PA. PAS staining, original magnification ×200.

In the diabetic apoE−/mice, the kidneys appeared to be unaltered. Examinations with light microscope revealed mesangium hypercellularity, no dular or diffuse MME, and rare foci of mesangiolysis in diabetic mice without PA treatment. On the other hand, diabetic mice treated with PA had significantly reduced MME compared with the diabetic untreated mice (Figure 3). There were no significant morphological changes observed in the glomeruli of the control mice either with or without dietary PA supplementation.

## 4. Discussion

Our current findings are in line with the previous observations that PA treatment attenuated both hyperglycemia in diabetes mellitus and DN [15]. We also report the novel observation that, regardless of the time of PA treatment (i.e., 1 month before, at the same time, or 1 month after induction of diabetes mellitus), PA supplementation significantly reduced hyperglycemia, especially in the pre-STZ group where hyperglycemia was confirmed before PA treatment. Our observations of increased inflammatory cytokine suggested that inflammation played an important role in the development and progression of DN. The obtained data strongly indicated a therapeutic effect of PA on diabetes mellitus.

The effects of dietary PA supplementation on hyperglycemia and hyperglycemia-induced nephropathy are not fully understood [12, 14, 30]. The hypoglycemic effect of PA could be attributed to different mechanisms that include increasing of insulin sensitivity in type-2 diabetic patients [31] and/or direct binding site at the tyrosine kinase domain of the insulin receptor [32].

The obtained data indicated the increase of oxidative stress in the diabetic mice. The oxidative stress markers like the elevated urinary 8-isoprostane, which is considered a reliable parameter of *in vivo* lipid peroxidation [33], increased kidney MDA, and the reduced GSH are significantly attenuated as a result of PA treatment. The obtained data are in line with other previous data [34]. The mitochondria are considered the major organelles which produce superoxide radicals. As result of the absence of both of the DNA repair systems and histones, the mitochondria are a primary target for oxidative damage [35]. Accordingly, superoxide released from mitochondria could be proposed as a mechanism for the development of diabetic complications [36]. Our data showed that the expression of SOD and its total activity in the kidney of diabetic mice were markedly reduced, whereas PA treatment restored SOD activity. Accordingly, the PA protection of the kidney from damage induced by the production of superoxide radicals occurs via strengthening of the antioxidant defense mechanisms of apolipoprotein E-deficient mice such as SOD.

The role of dietary PA supplementation in the attenuation of AGEs formation *in vivo* is not fully understood. Our hypothesis is that PA inhibits AGEs production in two ways. PA improved glucose metabolism and reduced methylglyoxal formation. Methylglyoxal is a dicarbonyl that is generated in glycolysis from glyceraldehyde-3-phosphate. Methylglyoxal plays a key role in the AGEs formation [37]. Treatment with PA resulted in increasing the uptake of glyceraldehyde-3-phosphate in the Krebs cycle and enhanced the generation of ATP [38]. This resulted in decreasing the amount of glyceraldehyde-3-phosphate available for methylglyoxal formation. 

It has been reported that AGEs trigger the activation of NF-*κ*B by interaction with the receptor of AGE (RAGE), leading to the translocation of NF-*κ*B to the nucleus where it induces transcription of its controlled inflammatory genes like IL-6, RAGE, TGF*β*, and so forth. Therefore, AGEs accumulation in the kidney has been regarded as an index of progressive renal damage in DN. RAGE is an important receptor for mediating AGE effects. Binding of AGEs such as CML to RAGE enhances oxidative stress in renal tissues. RAGE signals via NF-*κ*B activate target genes which have harmful potentials for the diabetic kidney. The higher levels of AGEs and IL-6, the overexpression of TGF*β* and RAGE, and the marked NF-*κ*B-p65 activation in the diabetic untreated animals showed a higher degree of oxidative stress and inflammation in the diabetic untreated animals. These elevated levels of AGEs, IL-6, RAGE, TGF*β*, and the significant NF-**κ**B-p65 activation were reduced as a result of PA administration. Thus, our data suggest that part of the beneficial effect of PA includes the disruption of the detrimental AGE-RAGE-NF*κ*B pathways.

PA feeding can improve the general health of the diabetic animals, including prevention of severe weight loss, suggesting that glucose metabolism is responsible for this reduction. PA is a naturally occurring antioxidant and could play an important role in the activity of several mitochondrial enzymes that are involved in the oxidation of glucose and ATP production. Therefore, the beneficial effects of PA on diabetes mellitus and DN could be attributed to the combined anti-inflammatory/antioxidant effects, the metabolic regulations that include increasing of the oxidation of glucose, and the attenuation of NF-*κ*B activation rather than its antioxidative effects alone. The obtained data were in line with those of previous studies [5, 7, 9, 15, 28, 30, 31, 37, 38].

## 5. Conclusion

The present study demonstrated that dietary supplementation of PA significantly improved hyperglycemia and renal function in apolipoprotein E-deficient mice. In addition, it attenuated NF-*κ*B activation and inflammation and improved kidney antioxidant status. PA is widely distributed in human diets, implying a lack of toxicity. PA, therefore, is a useful dietary adjunct for the treatment of diabetes mellitus and its complications. It is also considered as an important source required for the discovery of new active antidiabetic agent(s). Further preclinical experiments into the utility of PA supplementation are needed.

## Figures and Tables

**Table 1:** Effects of STZ treatment and dietary PA feeding on the food intake, bodyweight, albuminuria, biochemical parameters, serum AGE, TNF*α*, IL-6, GSH, and 8-hydroxy-2′-deoxyguanosine of apolipoprotein E-deficient mice.

	Control	Control + PA	Diabetic	Diabetic + PA
Initial bodyweight, g	195.13 ± 9.5	196.33 ± 6.5	196.44 ± 7	197 ± 7.2
Final bodyweight, g	265.5 ± 8.2	234 ± 12.3^a,b^	161.8 ± 7.2^a^	215.52 ± 8.9^a,b^
Glucose, mg/dL	92.78 ± 0.45	96.21 ± 0.57^a,b^	265.35 ± 1.45^a^	106.71 ± 1.23^b^
HbA1c, %	5.24 ± 0.41	6.88 ± 0.52^b^	9.42 ± 0.34^a^	6.85 ± 0.38^b^
Albumin, g/L	38.4 ± 5.7	38.9 ± 8.1	38.2 ± 7.7	38.7 ± 7.5
Total protein, g/L	76 ± 7	69.91 ± 7.5^b^	62 ± 6.5^a^	65.6 ± 7.1
GSH, mmol/L	0.281 ± 0.015	0.172 ± 0.02^a,b^	0.105 ± 0.03^a^	0.145 ± 0.021^a,b^
Catalase, U/g Hb	94.54 ± 14	86.55 ± 12^b^	50.98 ± 15^a^	70.11 ± 11^a,b^
Glutathione reductase, U/gHb	4.25 ± 0.09	3.21 ± 0.94^b^	2.25 ± 0.62^a^	2.54 ± 0.54^a,b^
Glutathione peroxidase, U/gHb	57.1 ± 11	69.9 ± 9.55^b^	145.5 ± 45^a^	89.54 ± 10.2^b^

Data shown represent mean ± SE of mice, 12 weeks after the treatment with STZ or with the buffer (control) in presence or absence of PA. ^a,b^Significance difference between groups.

**Table 2:** Oxidant/antioxidant parameters as well as concentration of carboxymethyl lysine (CML) in rat retinas.

	Control	Control + PA	Diabetic	Diabetic + PA
MDA, nmol/mg protein	2.45 ± 0.16	2.95 ± 0.15^b^	4.75 ± 0.17^a^	2.95 ± 0.15^b^
GST, nmol substrat·mg protein^−1^·min^−1^	176 ± 31	151 ± 9^a,b^	82 ± 13^a^	151 ± 9^a,b^
GSH-Px, nmol substrat·mg protein^−1^·min^−1^	0.92 ± 0.17	0.82 ± .165^b^	0.34 ± 0.09^a^	0.82 ± .165^b^
Catalase, IU·mg protein^−1^	2.61 ± 0.032	1.45 ± 0.21^a,b^	0.52 ± 0.03^a^	1.45 ± 0.21^a,b^
SOD, nmol substrat·mg protein^−1^·min^−1^	3.53 ± 0.45	2.95 ± 0.75^b^	1.82 ± 0.35^a^	2.95 ± 0.75^b^
GSH, nmol/mg protein	16 ± 3	16 ± 2.3	16.11 ± 2	16 ± 2.3
CML, pg/mg protein	3.54 ± 0.22	3.2 ± 0.6^b^	8.81 ± 0.34^a^	3.2 ± 0.6^b^

Oxidative stress markers in the kidney homogenate of apoE−/mice. Mice treated either with or without STZ in presence or absence of PA. PA treatment was continued for 12 weeks after the onset of diabetes. Data shown represent mean ± SEM. ^a,b^Significance difference between groups.
